# Significant Near-Field Enhancement over Large Volumes around Metal Nanorods via Strong Coupling of Surface Lattice Resonances and Fabry–Pérot Resonance

**DOI:** 10.3390/ma15041523

**Published:** 2022-02-18

**Authors:** Yunjie Shi, Yuming Dong, Degui Sun, Guangyuan Li

**Affiliations:** 1 Schools of Science, Changchun University of Science and Technology, 7089 Weixing Road, Changchun 130022, China; yj.shi@siat.ac.cn; 2 CAS Key Laboratory of Human-Machine Intelligence-Synergy Systems, Shenzhen Institute of Advanced Technology, Chinese Academy of Sciences, Shenzhen 518055, China; ym.dong@siat.ac.cn; 3 Shenzhen College of Advanced Technology, University of Chinese Academy of Sciences, Shenzhen 518055, China

**Keywords:** field enhancement, strong coupling, plasmonic–photonic coupling system, surface lattice resonance, Fabry–Pérot resonance

## Abstract

Metal nanoparticles supporting plasmons are widely used to enhance electromagnetic fields, resulting in strong light–matter interactions at the nanoscale in a diverse range of applications. Recently, it has been shown that when metal nanorods are periodically arranged with proper lattice periods, surface lattice resonances (SLRs) can be excited and near fields can be greatly enhanced over extended volumes. In this work, we report significant near field enhancement over even larger volumes by placing the metal nanorod array within a Fabry–Pérot (F-P) microcavity. Simulation results show that by taking advantage of strong coupling between the SLR and the photonic F-P resonances, the electric field intensity of the bonding split mode can be enhanced by up to 1935 times, which is about three times of the enhancement of the SLR, and the greatly enhanced field can extend over most of the F-P microcavity. We further show that the F-P resonances of both odd and even orders can strongly couple to the SLR by varying the nanorods position from the middle of the microcavity. We expect that the proposed plasmonic-photonic coupling system will find promising applications in nanolasers, nonlinear optics and sensing.

## 1. Introduction

Metal nanoparticles can support localized plasmon resonances, which strongly confine light to the nanoscale regions around the metal nanoparticles, resulting in greatly enhanced near fields [1,2]. This will lead to enhanced light–matter interactions at the nanoscale and find promising applications [3] in nanolasers [4], strong coupling [5], nonlinear optics [6], emission enhancement and sensing [7]. However, localized plasmon resonances suffer from relatively high loss (including absorption loss and radiation loss) [8], which results in broad linewidth and relatively small quality factor, and highly confined fields, which indicate that the enhanced light–matter interactions only occur in the close vicinity of metal nanoparticles. These drawbacks pose limitations on the performance of the related nanoscale devices. For example, based on these restrictions, Li et al. [9] investigated fundamental limitations to the ultimate Kerr nonlinear performance of plasmonic waveguides and provided insight into the modest nonlinear performance of a nonlinear dielectric waveguide doped with silver nanoparticles [10].

In order to alleviate these drawbacks, metal nanoparticles are periodic organized such that surface lattice resonances (SLRs) can be excited. Compared with localized plasmon resonances, SLRs have many attractive merits including narrow linewidths and high quality factors, and strong field enhancement extended over large volumes [11,12,13]. These characteristics make metal nanoparticles that support SLRs an excellent platform for light–matter interactions on the nanoscale [11]. For example, SLR-based nanolasers [14,15,16] and nonlinear optical devices [17,18,19] have shown superior performance recently. Nevertheless, open questions arise such as how to further increase the field enhancement of the SLR, and is it possible to extend the enhanced near fields over even larger volumes.

One approach to further enhance the near fields around metal nanoparticles is to couple or hybridize the modes involved, which makes use of Fano resonances [20], electromagnetically induced transparency [21], or strong coupling effects [5]. For example, by placing metal nanoparticles within or close to microcavities, which confine the electromagnetic field within a very small volume and allow large field enhancement, the localized fields around metal nanoparticles can be greatly enhanced [22], and the sensing properties of a localized plasmon sensor can be improved [23]. Quite recently, the authors of [24] proposed the strong coupling between the SLR and photonic Fabry–Pérot (F-P) resonances by placing a metal–insulator–metal nanopillar array within an F-P microcavity formed by a thin film and an air/SiO${}_{2}$ interface. However, because of the low reflectance at the air/SiO${}_{2}$ interface, the electric fields of the SLR are not enhanced.

In this work, we place a two-dimensional (2D) array of gold nanorods in an F-P microcavity formed by two thin metal film. We will numerically show that the SLR supported by the 2D gold nanorod array can strongly couple to the photonic F-P resonances. This can result in significantly enhanced electric fields over very large volumes around the gold nanorods: the maximum electric field intensity enhancement reaches 1935 times, which is about three times of that of the SLR; the enhanced electric fields can extend over most region of the F-P microcavity with length 0.765 μm. We will also show that both the odd and even F-P resonances can strongly couple to the SLR. We therefore expect this plasmonic–photonic strong coupling system will find potential applications when enhanced light–matter interactions over large volumes are required.

## 2. Structure Design and Numerical Setups

**Figure 1:**
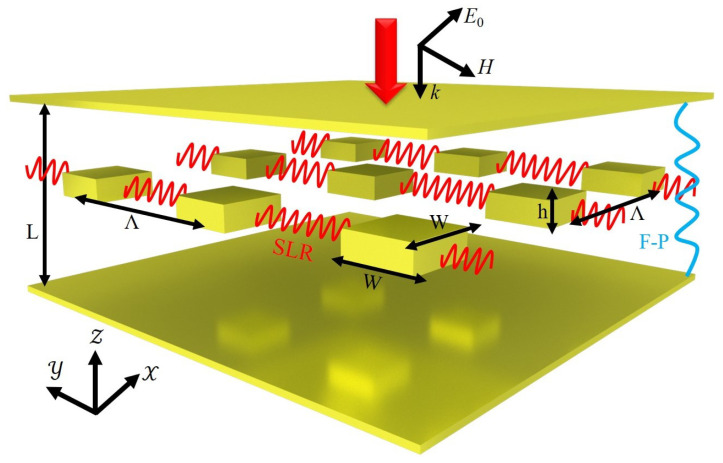
Schematic of the strong coupling between the F-P and the SLR resonance when metal nanorods are positioned within an F-P cavity formed by two gold mirrors.

Figure 1 illustrates the proposed plasmonic-photonic coupling system composed of an F-P microcavity formed by two thin gold films, and a two-dimensional infinite periodic array of gold nanorods embedded in the F-P microcavity. The gold films have thickness of 20 nm and are separated by distance *L*. The gold nanorods have thickness *h*, period Λ in both *x* and *y* directions, and square-shaped cross section with side length *W*. The F-P cavity is filled by silica with the refractive index of $n_{0}=1.45$, and the substrate and superstate are also taken to be silica. Unless otherwise specified, the gold nanorods have a square cross section with side length w=400 nm, thickness h=150 nm, and periods Λ=1300 nm in both *x* and *y* directions. The plasmonic–photonic coupling system is normally illuminated by plane wave polarized along the *x* direction.

We simulated the reflectance spectra and the near-field distributions of the proposed structure, the gold nanorod array, and the unperturbed F-P microcavity with a home-developed package for the fully vectorial rigorous coupled-wave analysis (RCWA), which was developed following [25,26,27]. In the simulations, the wavelength dependent permittivities of gold were taken from in [28].

## 3. Results and Discussion

### 3.1. Nanorods in the Middle of Microcavity

**Figure 2:**
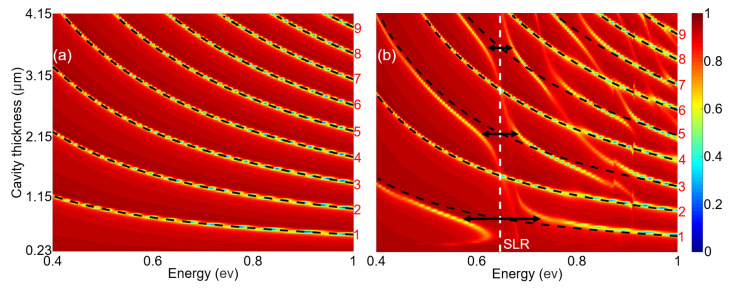
Simulated reflectance spectra of (**a**) the unperturbed F-P cavity and (**b**) the proposed structure formed by periodic metal nanorods placed in the middle of the microcavity with different cavity lengths. The F-P resonances are indicated by black dashed curves in (**a**,**b**), and the SLR wavelength is indicated by the vertical white dashed line in (**b**).

Figure 2a depicts the simulated reflectance spectra of the unperturbed F-P microcavity with different lengths ranging from L=230 nm to L=4.15μm. The resonances of different orders (indicated by the numbers *N* on the side) are indicated by black dashed curves. Indeed, the wavelengths of these F-P modes can be calculated by

(1) $$\begin{array}{c} \lambda_{N}=\frac{2n_{0}L}{N-\Delta \phi /\pi }\;, \end{array}$$

where *N* is the order of F-P mode, Δϕ is the reflection phase by the thin metal mirror.

These photonic F-P resonances supported by the microcavity and the SLR supported by the gold nanorod array are depicted as black dashed curves and a white dashed line in the dispersion diagram of the proposed structure, as shown in Figure 2b. Here, the nanorods are placed in the middle of the F-P microcavity. It is evident that the interactions between the F-P resonances of odd orders and the SLR result in anticrossings of these modes. The splitting widths are much larger than the coupled resonance linewidths, confirming that these interactions are in the strong coupling regime. For the F-P resonances of even orders, no anticrossing can be observed, suggesting that these photonic resonances are completely unperturbed by the SLR. We emphasize that these behaviors are similar with those of the strong coupling between localized plasmons and the F-P resonances [5,22,29].

**Figure 3:**
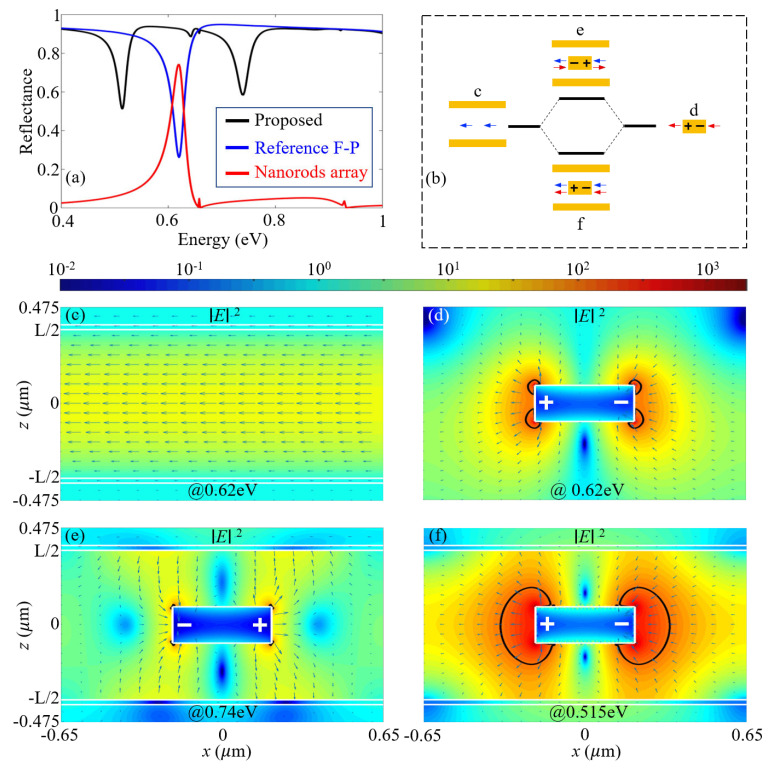
(**a**) Simulated reflectance spectra of the proposed plasmonic–photonic system (black curve), the reference F-P cavity (blue curve) and the nanorods array (red curve). The calculations were performed with L=765 nm. (**b**) Schematic illustration of the strong coupling of the first-order F-P resonance and the SLR into a bonding and an antibonding mode. The arrows indicate the directions of the near-field electric fields. (**c**–**f**) Simulated electric field distributions ${\left|E\right|}^{2}$ (color for intensity and arrows for directions) in the unit cell of (**c**) the reference first-order F-P resonance, (**d**) of the SLR of the nanorods array at 0.62 eV, and (**e**,**f**) of the split modes of the proposed structure that are resonant at (**e**) 0.74 eV and (**f**) 0.515 eV. The nanorods array and the thin metal films are outlined by white lines. “+” and “−” in (**d**–**f**) indicate charge distributions. The black curves in (**d**–**f**) show contours for electric field intensity enhancement of 100.

We first examine the anticrossing due to the strong coupling between the first-order F-P resonance and the SLR. Figure 3a depicts the simulated reflectance spectra of the gold nanorod array (blue curve), the unperturbed F-P microcavity (red curve) and the proposed plasmonic-photonic coupling system (black curve) with L=765 nm. Results show that SLR is excited in the gold nanorod array and the first-order F-P resonance is excited in the unperturbed microcavity at 0.62 eV (or 2 μm). For the proposed structure, it is observed the photonic mode is split into two modes, which are resonant at 0.74 eV and 0.515 eV, and the splitting energy is 225 meV. This corresponds to 36% of the resonant energy, further validating that we are in the strong coupling regime.

The simulated near-field plots in Figure 3c–f provide a deeper insight concerning the nature of these split modes. Figure 3c shows that the electric field of the first-order F-P resonance is strongest at the center, and the field enhancement factor is only 15. Figure 3d shows that dipolar oscillations are excited in the gold nanorod, and the electric field is greatly enhanced over extended volumes. The maximum field enhancement factor reaches as high as ${\left|E\right|}^{2}/{\left|E_{0}\right|}^{2}=680$, and the enhanced fields with enhancement factor above 100 (enclosed by the black contours) extend over tens of nanometers away from the gold corners. These are typical features of the dipolar SLR. When the gold nanorod array is placed in the middle of the F-P microcavity, Figure 3e shows that for the high energy hybridized mode at 0.74 eV, the antibonding characteristic significantly suppresses near fields. Remarkably, for the low-energy hybridized mode at 0.515 eV, Figure 3f shows that the bonding characteristic significantly enhances near fields extending over even larger volumes compared with the SLR fields in Figure 3d. In this scenario, the maximum field enhancement factor now reaches up to 1673, which is 2.5 times that for the SLR. Moreover, the enhanced fields with enhancement factors above 100 now extend over a large volumes covering ∼200 nanometers away from the gold corners. The bonding and antibonding characteristics of the split modes in Figure 3e,f are better visualized in Figure 3b.

**Figure 4:**
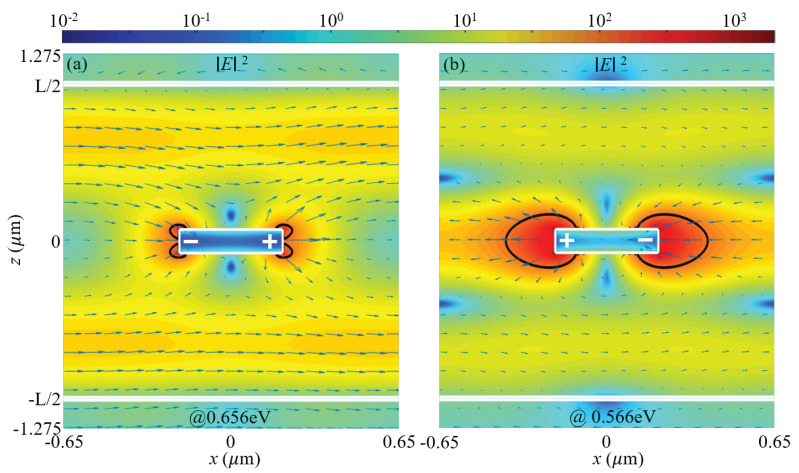
Similar to Figure 3e,f but for the split modes due to the strong coupling between the SLR and the third-order F-P resonance. These modes are resonant at (**a**) 0.656 eV and (**b**) 0.566 eV. The calculations were performed with L=2147 nm.

For the strong coupling between the SLR and the third-order F-P resonance with L=2147 nm, and the splitting energy is 90 meV, as shown by Figure 2b. In Figure 4, we plot the electric field distributions of the split modes that are resonant at 0.656 eV and 0.566 eV. Results show that for the antibonding mode at high energy, the near fields are greatly suppressed; whereas for the bonding mode at low energy, the near fields are significantly enhanced and extended over large volumes. These characteristics are similar to the split modes in Figure 3e,f. Additionally, the extended near fields around the gold nanorods are restricted to the central cavity mode defined by the two node planes of the standing wave in the F-P microcavity.

**Figure 5:**
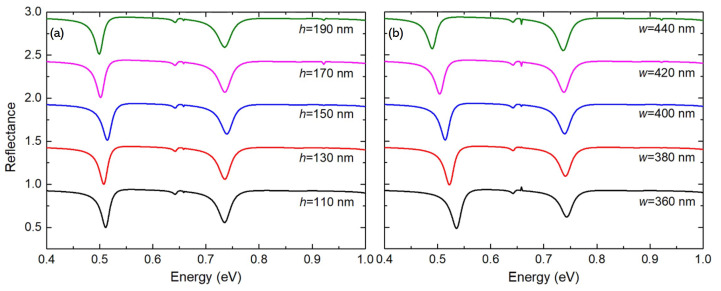
Simulated reflectance spectra of the proposed plasmonic-photonic system with different (**a**) nanorod heights *h* and (**b**) side widths *w*.

Additionally, we investigated the effects of the structural size *w* and *h*, which may be imperfect during the fabrication. Figure 5a shows that as the nanorod height increases from h=110 nm to 190 nm, the low-energy hybridized mode experiences more redshift (shift to the low energy side) than the high-energy mode. This results in that the splitting energy increases slightly. Figure 5b shows that, as the nanorod side width increases from w=360 nm to 440 nm, the low and high energy hybridized modes, and thus the splitting energy share similar behaviors as *h* increases. Therefore, thanks to the large tolerance to the fabrication imperfections, which will greatly facilitate the design and nano-fabrication, we expect the simulated results in this work can be conveniently observed in experiments.

### 3.2. Nanorods Away from the Middle of Microcavity

**Figure 6:**
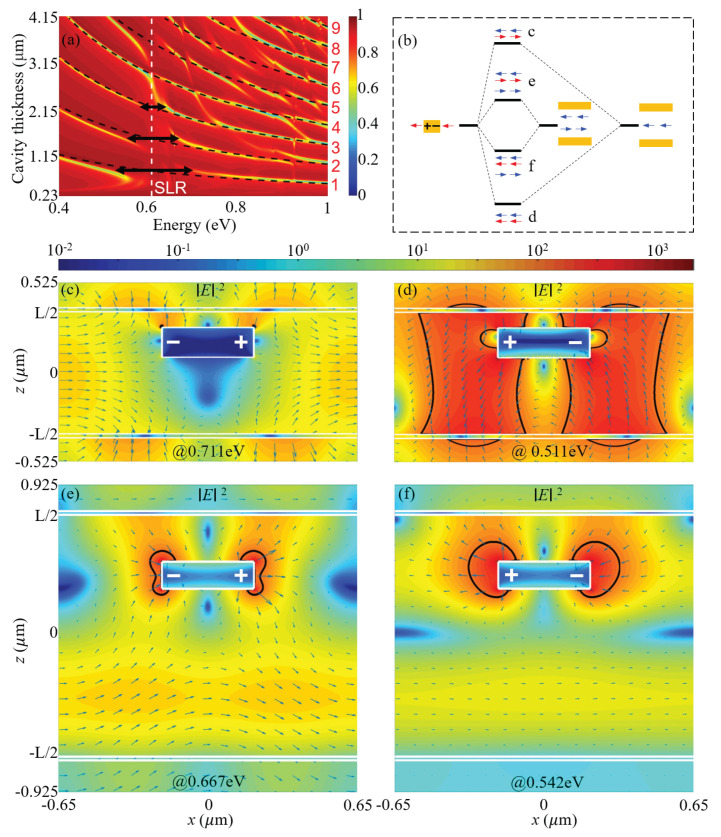
Strong coupling between the SLR and the F-P resonance when the gold nanorod array is positioned with distances to the top and bottom gold mirrors with a ratio of 1:3. (**a**) Simulated reflectance spectra for different cavity lengths. The F-P resonances are indicated by black dashed curves, and the SLR wavelength is indicated by the vertical white dashed line. (**b**) Schematic illustration of the strong coupling of the first- and second-order F-P resonances and the SLR into bonding and antibonding split modes. (**c**–**f**) Simulated electric field distributions ${\left|E\right|}^{2}$ of the split modes at (**c**) 0.711 eV (**d**) 0.511 eV due to the strong coupling between the SLR and the first-order F-P resonance with L=765 nm, and at (**e**) 0.667 eV and (**f**) 0.542 eV due to the strong coupling between the SLR and the second-order F-P resonance with L=1490 nm. The nanorods array and the thin metal films are outlined by white lines. “+” and “−” in (**d**–**f**) indicate charge distributions. The black curves in (**d**–**f**) show contours for electric field intensity enhancement of 100.

We now discuss the effects of the vertical position of the periodic nanorods. As an example, we position of the gold nanorods in such a way that the ratio of the distances to the two gold cavity mirrors are 1:3. Figure 6a shows that the SLR can strongly couple with the F-P resonances of both odd and even orders. This behavior is distinct from that of the strong coupling between the F-P resonance and the localized plasmons [5,22]. As the F-P resonance order increases, the splitting energy decreases in general. For the first- and second-order F-P resonances with L=765 nm and L=1490 nm, the splitting energies are 200 meV and 125 meV, which are 32% and 20% of the resonant energy, respectively. These results confirm the strong coupling regime. The corresponding electric fields of the split modes due to the strong coupling are shown in Figure 6c–f, respectively. Similar to the above results shown in Figure 3e,f and Figure 4, the antibonding modes that are resonant at high energies have suppressed fields around the metal nanorod, whereas the bonding modes that are resonant at low energies have significantly enhanced electric fields over large volumes; the extended near fields around the gold nanorods are restricted to one cavity mode defined by the two node planes of the standing wave in the F-P cavity. The bonding and antibonding characteristics of the split modes in Figure 6c–f are illustrated in Figure 6b.

Strikingly, Figure 6d shows that for the bonding split mode, the largest field intensity enhancement reaches 1935, which is about three times of that of the SLR; the electric fields in most regions of the F-P microcavity with length L=765 nm are enhanced by more than 100 times. This region of greatly enhanced field is much larger than those of the SLR in Figure 3d and of the split modes in Figure 3f and Figure 4b. Therefore, we show that by placing the nanorod array in the F-P microcavity, regardless of the position, the field enhancement of the bonding split mode can reach twice or three times of that of the SLR, and the enhanced fields can be greatly extended to an extent of filling most regions of the F-P microcavity.

## 4. Conclusions

In conclusion, we have numerically shown that strong coupling between the SLR and the F-P resonances of odd orders can be observed when the gold nanorod array is placed in the middle of the F-P microcavity. The splitting energy reaches as high as 225 meV, which is 36% of the resonant energy. For the bonding split mode at low energy, the electric fields can be enhanced by 1673 times, which is 2.5 times of those of the SLR; the enhanced fields extend over ten times larger volumes compared with the SLR. When the gold nanorod array is placed in such a way that the ratio of the distances to the two gold cavity mirrors are 1:3, results have shown that the SLR can strongly couple with the F-P resonances of both odd and even orders. The maximum electric field enhancement factor can reach 1935, which is three times of that of the SLR. Strikingly, the enhanced electric fields can extend over most regions of the F-P microcavity with length 765 nm. We therefore expect that this work will provide a new approach for greatly enhancing light–matter interactions over extending volumes, and that the proposed plasmonic-photonic coupling system will find promising applications especially in emission enhancement, nonlinear optics, nanolasers and ultrasensitive sensing.

## Data Availability

The data presented in this study are available on request from the corresponding author.
